# Workplace bullying and its preventive measures and productivity among emergency department nurses

**DOI:** 10.1186/s12913-019-4268-x

**Published:** 2019-07-03

**Authors:** Suhair Hussni Al-Ghabeesh, Haya Qattom

**Affiliations:** Faculty of Nursing. Head of the Clinical Nursing Department, Al-Zaytoonah University of Jordan, Airport Street, Amman, Jordan

**Keywords:** Emergency department, Jordanian nurses, Productivity, Workplace bullying

## Abstract

**Background:**

Workplace bullying has adverse effects on nurses’ productivity and emotional well-being and increases nurses’ desire to leave their jobs. Bullying is a common phenomenon that has been reported worldwide. Emergency Department (ED) nurses are particularly exposed to bullying as a result of their job stressors and demands.

**Purposes:**

To examine the prevalence of bullying and the impact of preventive measures on productivity among Jordanian ED nurses; and to examine bullying in relation to personal and organizational factors.

**Methods:**

We surveyed ED nurses in five hospitals in Amman, Jordan – two government hospitals and three private hospitals. The eligibility criteria for the study, met by 134 persons, were having at least an associate degree and having worked in the ED for at least six months. We used a four-part questionnaire that included demographic data, the Negative Acts Questionnaire, questions on prevention of bullying, and a health and productivity survey. Data analysis included descriptive and inferential statistics.

**Results:**

A total of 120 ED nurses joined the study, an 89.6% response rate. The majority of participants were male (65%) and their mean age was 29.4 years. Ninety percent of the participants reported being bullied. Nurses with less experience in the ED were exposed to more bullying compared to other nurses. Of nurses who reported being bullied, 61.7% reported associated decreased productivity, including the ability to respond to cognitive demands, provide support, appropriate communication, safe care, and competent care. The overall mean score for the prevention of bullying questionnaire was 94.51 out of 168 (SD = 23.43). Drilling down, the highest mean score was for the “Individual sub-scale”, and the highest item mean score was for “*I know the process of how to report bullying”*.

**Conclusion:**

Bullying is prevalent among ED nurses in Jordan; it has significantly influenced the nurses’ perception of their productivity and the quality of care they provide. Although nurses reported adopting measures to prevent bullying, they were insufficient to address this widespread problem.

**Implications for nursing and health policy:**

Bullying is a common occurrence in nursing practice in Jordan, as in other places. It has a detrimental effect on the quality of health care. Accordingly, interventions, which we describe, should be undertaken to minimize the incidence and impact of bullying.

## Background

Workplace bullying (WPB) is a major public problem that has received growing attention and has become an international problem documented in a number of countries within a diversity of professions [1]. WPB against emergency department (ED) nurses is considered one of the most common and widespread types of hospital-based violence [1]. There is also some evidence that WPB adversely affects the quality of nursing care [2].

A priority of nurse leaders and managers is to attend to the problem of bullying experienced by nursing staff. Bullying is normally not about a single isolated event but, rather, about a pattern of behaviors that are repeatedly and persistently directed towards one or more employees [3]. WPB is divided into four types, as described in Table 1 and 2:

**Table 1:** Types of bullying

Type of bullying	Description
Type I, criminal intent	occurs where the perpetrator has no legitimate relationship to the business or its employees
Type II	occurs where the perpetrator, a customer, client, or patient, becomes violent while receiving a service through the workplace
Type III	involves employee-to-employee incidents where the perpetrator is a current or previous employee
Type IV	occurs when the perpetrator has a personal relationship with the employee but does not have an association with the workplace

**Table 2:** Demographic Profile of the Participants (*N* = 120)

Variable	Mean (SD)	Range	N (%)
Gender of the Attacker			
Male			63 (52.5)
Female			21 (17.5)
Both			36 (30)
Experience in ED (year)	4.0 (3.2)	1–13	
Experience in nursing (year)	6.7 (4.6)	1–28	
Frequency of attack	2.0 (2.1)	1–21	
Experience in hospital (year)	3.8 (3.4)	1–15	
Did you have a friend relationship with the attacker?			
Yes			34 (28.3)
No			86 (71.7)
Organizational tolerance and reward of bullying			
Yes			22 (18.3)
No			98 (81.7)
Type of hospital			
Private			60 (50)
Governmental			60 (50)
Misuse of organizational processes/procedures			
Yes			39 (32.5)
No			81 (67.5)

In the current study, we focused on Type III bullying, also known as “Lateral Violence” [4]. Type III bullying involves behaviors occurring between employees in which the perpetrator is a current or past worker of the workplace. The perpetrators of Type III bullying usually display bullying that is verbal or psychological, and only less frequently does it consist of physical abuse [5]. Type III bullying is the most widespread type of workplace bullying experienced by nurses. WPB includes behaviors that are obvious and behaviors that are concealed. Obvious behaviors associated with Type III WPB include shouting, name-calling, pushing, or physically overcrowding someone’s path. The more complicated behaviors associated with WPB are relatively concealed. These include behaviors such as withholding information, tattling, excessively supervising work or assigning an irrational workload from supervisors [2]. The ten most common forms of WPB behaviors among nurses are: non-verbal innuendo, verbal insult, undermining activities, withholding information, sabotage, infighting, gloating, backstabbing, failure to respect privacy, and broken confidences [6]. Workplace bullying is a serious problem among registered nurses. Up to 40% of nurses are exposed to bullying behaviors at work, including exclusion, intimidation, and belittlement [7], on a regular basis [8–10].

Researchers have confirmed that bullying has negative effects at the individual and organizational levels [11, 12]. Because of those effects, some organizations such as the American Nursing Association (ANA) have created statements on incivility, violence, and workplace bullying [13]. At the individual level, bullying leads to elevated levels of work-related health problems such as stress, anxiety, depression, sleep problems and irritability [12].At the organizational level, there is a decrease in nurses’ productivity [13] and an increase in their absenteeism and use of sick leave. This ultimately results in substantial costs to the hospital: it will pay for nurses during their sick leave and also for the costs of having personnel officers, personnel consultants, and various managers to handle the situation. Additionally, the hospital will pay for temporary nurses who will replace nurses who are absent or on sick leave. Another organizational price of bullying is an increased rate of turnover of qualified nurses, which can lead to a decline in patient safety [14, 15].

Prior to this investigation, most studies conducted in Jordan limited their focus to violence (actions or words from patients or their family members that are intended to hurt nurses) in the ED and used the term “bullying” incorrectly [16–18]. Thus, a comprehensive understanding of bullying, particularly Type III bullying in the ED had not yet been obtained. More information about this dangerous phenomenon and its prevalence was needed. With this study, we sought gain new information that might influence the development and use of preventive measures to reduce bullying in EDs in Jordan and around the world.

Delayed understanding has made it difficult for nursing professionals to recognize bullying, react to it appropriately, and, ideally, prevent it. The aims of this study were to: 1) describe the incidence of bullying; 2) explore the effects of bullying on nurses’ productivity; and 3) examine nurses’ perceptions of the nature and effectiveness of measures taken to prevent bullying in the ED.

## Methods

### Study design

A cross-sectional, descriptive, correlational design was used to collect data from ED nurses in Amman, Jordan using a self-administered questionnaire. Data were collected regarding workplace bullying, productivity, preventive measures, and demographic and organizational characteristics.

### Sampling

The study took place over a period of 4 months from 10 April to 10 August 2017. There are two governmental hospitals in Amman, Jordan, that have large EDs, and both participated. There are also six private hospitals with large EDs in Amman, and three of those agreed to participate. There are 500 nurses working in the EDs of the five participating hospitals. Of these, 134 met the inclusion criteria for the study by having at least an associate degree in nursing and having worked in the ED for at least 6 months. The study questionnaires were given to all of them. Of the remaining 366, 250 have at least an associate degree but have worked in the ED for less than 6 months; and 116 do not have an associate degree.

### Ethical considerations

The approval of the Institutional Review Board at Al-Zaytoonah University of Jordan was obtained (reference number: 2017–2016/591/11). Also, ethical approvals were obtained from the Ministry of Health (MOH) and the three participating private hospitals.

### Informed consent and distribution of questionnaires

The principal investigator approached eligible participants individually, invited them to participate, and explained the purpose of the study. Participants were informed that their participation was voluntary and that they also had the right to terminate their participation at any time without giving any reason and without this decision affecting their work. Participants were also assured that their responses would be treated confidentially and without disclosure of their identity. Written informed consent was obtained from all participants.

### Study instruments

Four instruments were used in this study as follows:

- A demographic information sheet, developed by the researchers, includes the participant’s age, gender, height, weight, level of education in nursing, years of experience working as a nurse, and length of time employed in the ED. Further information was collected about organizational factors such as type of hospital, the type of shift nurses worked, and other variables related to bullying such as having been bullied or observed bullying in the past 6 months.
- The Negative Act Questionnaire-Revised (NAQ-R), is a standardized instrument with 23 items that assess perceived experiences of bullying at work [19]. Every item is written in behavioral terms, and the word bullying is not used until the last question. The measure uses a five-point Likert scale response option for the first 22 items to assess the frequency of exposure. The NAQ-R has shown good internal consistency with Cronbach’s alpha of 0.90 [14].
- The Healthcare Productivity Survey (HPS) is a 29-item scale with four subscales. It was developed to measure the perceived change in work productivity after exposure to a stressful event. The four subscales include: Cognitive Demands, Workload Demands, Support and Communication Demands, and Competent and Safe Care Demands. Demonstrated psychometric properties include strong content and construct validity for the four subscales [20].
- The Prevention of Bullying Questionnaire (PBQ) is a 42-item scale that assesses the respondent’s perception of the use of prevention measures. The scale contains three subscales: institutional prevention (7 items), unit prevention (19 items), and individual prevention (16 items) assessed using a 4-point Likert scale. The instrument was developed by Ganz, et al. [21], using a focus group of approximately ten ICU nurses. Cronbach’s α reliability for the entire scale in Ganz’s study was 0.89. Cronbach’s α reliabilities for each subsection were 0.88 (unit prevention), 0.90 (institutional prevention), and 0.41 (personal prevention).

### Data management and analysis

Data were analyzed using SPSS version 22.0. Descriptive statistics including frequencies, percentages, means, and standard deviations were used to describe the sample characteristics and all questions related to WPB among nurses. Pearson’s product moment correlation coefficient was used to examine the relationship between total scores of the intensity of bullying, age, and years of experience. An independent samples t-test was used for the variables with two categories (i.e. gender and type of hospital), and one way ANOVA was used for the variables with more than two categories (i.e. educational level and working shift) to examine the relationship between total scores of intensity of bullying and demographic characteristics including gender, marital status, level of education, and position, as well as for other recorded variables related to bullying, as mentioned above.

## Results

### Sample characteristics

A total of 120 out of 134 distributed questionnaires were retrieved, representing an 89.6% response rate. Seventy-eight participants (65%) were male; and 42 participants (35%) were female. The mean age for participants was 29.4 years (SD = 4.6), ranging from 22 to 50 years. The mean height of the participants was 171.3 cm (SD = 8.3) and their mean weight was 74.9 kg (SD = 14.67). Sixty-eight (56.7%) were married; 44 (36.7%) were single; and eight (6.7%) were either divorced or widowed. Most participants (*n* = 81, 67.5%) had a bachelor’s degree in nursing, and most worked as registered nurses (*n* = 88, 73.3%). The majority of the participants (*n* = 68, 56.7%) worked in rotating shifts with the A shift being from 7 am to 3 pm, the B shift from 3 pm to 11 pm, and the C shift from 11 pm to 7 am. Only 14 participants (11.6%) reported that they had specific training about how to deal with WPB. Among the participants, 52.5% reported that the perpetrators were males only, 17.5% reported females only, and 30% reported perpetrators from both sexes. Of the perpetrators, 33.3% were reported to be registered nurses; 30.0% were nurses working in the same unit; 53.3% were between ages 31–40; and 71.7% had no previous friendly relationship with the victim. The highest percentage of WPB occurrences were during the B shift (35.8%), then on the A shift (27.5%), and then on C shift (25.0%). The remaining participants (11.7%) stated that they were bullied on all shifts. A total of 63 participants (52.5%) witnessed acts of bullying at workplace, but only 13 (10.8%) participants reported that they, themselves, harmed a colleague emotionally. Only 22 participants (18.3%) think that their organization is concerned about WPB, and 39 participants (32.5%) think staff misuse regulations concerning bullying at workplace.

### Prevalence of workplace bullying

In response to the question “please state whether you have been bullied at work over the last six months,” 90% of the participants (*n* = 108) considered themselves victims of bullying; and, of these, only one (0.93%) reported being bullied daily, eight (6.6%) reported being bullied weekly, 33 (30.6%) reported being bullied now and then, and 66 (61.1%) reported being bullied rarely. Table 3 provides a complete description of each item of the NAQ-R.

**Table 3:** The mean and standard deviation for NAQ-R items (5-point Likert scale)

	Mean	*SD*
1- Someone withholding information which affects your performance (Work-related bullying)	1.766	.895
2- Being humiliated or ridiculed in connection with your work (Person-related bullying)	1.966	.986
3- Being ordered to do work below your level of competence (Work-related bullying)	1.858	.928
4- Having key areas of responsibility removed or replaced with more trivial or unpleasant tasks (Person-related bullying)	1.925	.997
5- Spreading of gossip and rumors about you (Person-related bullying)	1.966	1.011
6- Being ignored or excluded (Person-related bullying)	1.883	.971
7- Having had insulting or offensive remarks made about your person, attitudes, or private life (Person-related bullying)	1.966	1.076
8- Being shouted at, or being the target of spontaneous anger (Physically intimidating bullying)	1.908	.995
9- Intimidating behaviors such as finger-pointing, invasion of personal space, shoving, blocking your way (Physically intimidating bullying)	1.925	1.070
10- Hints or signals from others that you should quit your job (Person-related bullying)	1.833	.955
11- Repeated reminders of your errors or mistakes (Person-related bullying)	2.200	1.192
12- Being ignored or facing a hostile reaction when you approach (Person-related bullying)	2.133	1.129
13- Persistent criticism of your errors or mistakes (Person-related bullying)	2.166	1.183
14- Having your opinions ignored (Work-related bullying)	2.158	1.209
15- Practical jokes carried out by people you don’t get along with (Person-related bullying)	2.050	1.158
16- Being given tasks with unreasonable deadlines (Work-related bullying)	2.116	1.182
17- Having allegations made against you (Person-related bullying)	1.933	1.098
18- Excessive monitoring of your work (Work-related bullying)	2.225	1.198
19- Pressure not to claim something to which by right you are entitled (Work-related bullying)	2.208	1.235
20- Being the subject of excessive teasing and sarcasm (Person-related bullying)	1.966	1.180
21- Being exposed to an unmanageable workload (Work-related bullying)	2.291	1.252
22- Threats of violence or physical abuse or actual abuse (Physically intimidating bullying)	2.016	1.130

The mean score of the NAQ-R was 44.47 (*SD* = 15.78), indicating the average bullying level as “sometimes bullied”. Additional analysis was conducted to describe the frequency and percentage of participants who were subjected to various categories of bullying. It was found that 30.8, 22.5, and 46.7% were categorized as “not bullied”, “sometimes being bullied”, and “victims of WPB,” respectively. The mean item score for the NAQ-R was 2.02 out of 5.

The highest mean score was reported for work-related bullying (*M* = 2.08, *SD* = 0.78), followed by person-related bullying (*M* = 1.99, *SD* = 0.73), while the lowest mean score was reported for physically-intimidating bullying (*M* = 1.95, *SD* = .83).Regarding the work-related bullying sub-scale, the highest item mean score was Item 21: “Being exposed to an unmanageable workload”, (*M* = 2.29, *SD* = 1.25), while the lowest item mean score was Item 1: “Someone withholding information which affects your performance”, (*M* = 1.77, *SD* = 1.90). Regarding the person related bullying sub-scale, the highest item mean score was Item 11: “Repeated reminders of your errors or mistakes,” (*M* = 2.20, *SD* = 1.19), while the lowest item mean score was Item 10: “Hints or signals from others that you should quit your job,” (*M* = 1.83, *SD* = 1.96). Finally, the highest item mean score for physically intimidating bullying sub-scale was Item 22: “Threats of violence or physical abuse or actual abuse,” (*M* = 2.01, *SD* = 1.13), while the lowest item mean score was Item 7: “Being shouted at, or being the target of spontaneous anger or rage,” (*M* = 1.90, *SD* = 1.99).

### Relationship between workplace bullying and work productivity

The majority of participants (61.7%) reported decreased productivity after the exposure to WPB while 36.7% reported increased productivity, and only 1.7% (2 nurses) reported no change in productivity. As presented in Table 4, the mean scores of the subscales and the total score of healthcare productivity survey (HPS)were negative, indicating a decrease in the perceived average productivity of the participants. The support and communication subscale had the greatest decrease in productivity (*M* = 1.92). This includes items such as “coordinate care of my patients with other employees”, collaborate with other staff in getting their work completed, control your emotional reactions while work with coworkers, answer questions from coworkers, communicate with other departments regarding patient care, and provide comprehensive information when transferring patients for “safe handoffs”. While the cognitive demands subscale had the lowest decrease in productivity (*M* = -1.19). This includes items such as “keep your mind on your work, think clearly when working, be careful when working, concentrate on your work, be attentive to details and initiate or start work activities”. The total score of the NAQ-R was negatively and significantly correlated with HPS total score (*r* = −.27, *p* < .05).

**Table 4:** The mean scores for each sub-scale of HPS

	N	Minimum	Maximum	Mean	*SD*
Cognitive demands	120	−10.00	10.00	−1.19	5.66
Workload demands	120	− 12.00	12.00	−1.83	6.57
Support and Communication	120	−12.00	12.00	−1.92	6.65
Safety and competency	120	−20.00	20.00	−1.38	11.12
Total Productivity	120	−58.00	58.00	−6.86	30.04

In addition, three of the subscales of HPS were negatively and significantly correlated with HPS total score including cognitive demands; *r* = − 0.22, *p* < .05, support and communication; (*r* = −.32, *p* < .05), and safety and competency (*r* = − 0.28, *p* < .05). However, although there was a negative relationship between workload demands and the total score of NAQ-R (*r* = − 0.17), this relationship was not statistically significant *p* = .06).

### Perceptions of Jordanian emergency nurses regarding the preventive measures of bullying

The total mean score for the prevention of bullying questionnaire (PBQ) was 94.51 out of a possible total of 168 (*SD* = 23.43). Table 5 shows the mean scores for the unit, individual, and institutional subscales of the PBQ as well as for the item within each subscale having the highest and lowest mean score.

**Table 5:** The scores of the prevention of bullying questionnaire sub-scales and items with the highest and lowest scores

Variable	Mean (*SD*)	Highest item mean score (*SD*)	Name of the item with the highest mean score	Lowest item mean score (*SD*)	Name of the item with the lowest mean score
Unit sub-scale	2.20 (.58)	2.53 (1.02)	Item 39: There is a feeling of collegial support on my unit	1.38 (.83)	Item 1: My unit encourages or allows bullying
Individual sub-scale	2.26 (.59)	2.50 (1.02)	Item 14: I know the process of how to report bullying	2.02 (1.06)	Item 4: The hospital administration is unable to manage conflict or manages it ineffectively
Institutional sub-scale	2.33 (.79)	2.48 (1.05)	Item 11: My hospital is ethically committed to fostering an anti-bullying work environment	2.20 (1.02)	Item 7: There are known standards in my hospital about bullying

### The influence of personal factors and organizational factors on bullying

The results of the independent samples t-test indicated that the mean score of NAQ-R was not significantly different based on gender of the participant, *t* (118) = 1, 81, *p* = 0.07 and type of hospital *t* (118) = − 1.68, *p* = 0.10. Additionally, the results of one-way ANOVA indicated that the mean score of the NAQ-R was not significantly different based on educational level, *F* (2,117) =2.39, *p* = 0.10, and working shift, *F* (3,116) =1.79, *p* = 0.15.

The Pearson’s product-moment correlation analysis indicated that the length of experience in the ED was positively and significantly correlated with bullying at work place (*r* = 0.20, *p* < .01); i.e., the fewer the years of experience working in the ED the more likely a nurse will experience, or be exposed to, bullying. None of the other examined variables, e.g., height, weight, age, and years of experience in nursing, was significantly correlated with bullying at work place.

## Discussion

The purpose of this study was to assess the prevalence of bullying among Jordanian nurses working in the ED and the relationship between WPB and work productivity and the perception of preventive measures. The influence of personal factors and organizational factors on bullying was also identified.

The findings of the current study show that WPB has a very high prevalence among Jordanian nurses working in EDs, i.e., 90%. This result is consistent with previous studies which reported a high level of violence in EDs in Jordan [22–25]. Although bullying has not been studied extensively in Arab countries, the results of this study are also consistent with the results of a study conducted in Saudi Arabia [26]. Furthermore, the results of this study are consistent with the results of most studies conducted worldwide [13, 27–31]. This high level of prevalence might be due to the stressful work environment and role conflict between nurses in the EDs in Jordan [22, 23] and other Arab countries [26]. Shafran et al., confirmed that emergency room nurses were more exposed to violence than nurses in the internal medicine departments [31]; and Vessey et al., have concluded that bullying is a learned behavior that is dependent on the work environment [32].

In the current study, 65% of the participants were male. The percentage of male nurses is less elsewhere in the hospital: For example, in the neonatal, pediatric, obstetrics and gynecology, and female medical and surgical departments, all the nurses are female. In the kidney dialysis unit, intermediate unit and operation departments, most of the nurses are female. On the other hand, in the male medical and surgical departments, all nurses are male. In most western countries where WPB has been studied, the majority of nurses are female; and the prevalence of WPB is also high [33]. Although Wang and Hsieh found that gender was a factor in WPB and they considered it as a social factor that influence the incidence of workplace bullying [34], we conclude that the bullies are not simply persons of one gender towards the opposite and it is not a dominant factor of bullying.

In the three sub-scales of the NAQ-R, the highest mean score was reported for work-related bullying, and this is consistent with the results of some previous studies [35, 36]. The highest item mean score in this category or subscale was for “Being exposed to an unmanageable workload.” This can be the nature of work in an ED. Other studies, not just in ED settings or in Jordan, have found that an excessive workload had a significant effect on nurses’ exposure to WPB behaviors [21, 37–39].

The lowest mean score in the work-related bullying category was “Someone withholding information which affects your performance.” Yet, this was the most frequent negative behavior in a study by Johnson and Rea [40]. Their study, in contrast to ours, was not limited to ED nurses. The lowest mean score in the category of person-related bullying was reported for physically intimidating bullying. This is in line with the findings in the study of Ganz and her colleagues [21].

Only 11.6% of the participants reported that they had specific training about dealing with WPB, and only 18.3% of the participants think that their organization is concerned about WPB. Also, more than half of the participants stated that they need training to deal with bullying incidents. Accordingly, we and others believe there is an essential need for hospitals in Jordan to have a training program in this area [23, 41]. Additionally, enhancing the performance capabilities of the staff and promoting their communication skills through training programs might contribute to minimizing the acts of workplace violence and their consequences on the staff.

Abu-ALRub and Al-Asmar, and Al-Azzam, et al., have found in their studies of workplace violence that 70% of the participants state having no knowledge of a clear institutional policy concerning physical and verbal violence in the workplace as well as inability to report violent acts [38, 41, 42]. Undoubtedly, the absence of clear policies and special training concerning violent acts intensifies the occurrence of the phenomenon in EDs.

In the demographic survey, nurses were asked about the perpetrator of the bullying in the workplace. About 33% claimed that nurses were the most common perpetrators of bullying. This is consistent with a previous study by Berry et al. [13].The Johnson and Rea study found that 50% of bullying was perpetrated by nursing managers, and 38% by nurse co-workers [40].This result is confusing: One would think that nurses with a higher educational level might receive more instruction on how to communicate with sub-ordinates and guide them. This seems not to be the case.

In the current study, exposure to bullying events was significantly related to a reported decrease in productivity in the areas of cognitive demands, safety and competency, and support/communication demands. These findings suggest that while ED nurses try to maintain their pace of work, they experience trouble balancing the cognitive, emotional, and safety demands needed to deliver appropriate caring for their patients.

Berry et al., also have found that a higher incidence of bullying reported by nurses was associated with greater impaired cognitive status, decreased productivity, and poorer handling of job workload [13].

Gates et al., like us, found that poorer support and communication were related to WPB [34]. They reported that nurses who are bullied become unable to communicate with patients and visitors, unable to provide emotional support, and often experience feelings of detachment from patients and nursing colleagues.

When safety and competency of a nurse are affected by bullying, the bullying can lead to the nurse’s committing errors, such as medication errors. This is consistent with Roche, et al., who reported that all types of violence were linked to late administration of medication [44]. Roche, et al., believed that the reason for their finding a non-significant relationship between workload demands and the total score of NAQ-R was related to the characteristics of ED nurses: ED nurses have been trained to provide care for patients often in very stressful situations that involve taking care of critically ill patients under extreme clinical pressures. Also, ED nurses work conscientiously and with strong attachment to their work in fast-paced environments. This is further supported by Gates, et al. who stated that exposure to violent events was significantly related to decreased productivity in the areas of Cognitive Demands and Support/Communication Demands [43]. Similarly, Yildirim and Yildirim found that the most common thing nurses did to escape from bullying was “to work more carefully to avoid criticism [45].” To summarize these findings, it appears that the more bullying experienced by ED nurses, the greater their difficulty in achieving three of the areas of productivity included in the Healthcare Productivity Survey (cognitive demands, support and communication, and safety and competency).

The areas of nurses’ job performance that were mostly affected by WPB were job motivation, energy level, and commitment to work. It is known that WPB behavior is associated with depression, work motivation, concentration of work, productivity, commitment to work, and poorer relationships with patients, managers, and colleagues [37].

At the beginning of the demographic questionnaire, when we asked about the exposure to bullying, 63 of the 120 participants (52.5%) reported witnessing attacks of bullying at the workplace; whereas, after bullying was defined formally in the NAQ-R, 90% reported witnessing bullying in the workplace. Simply asking about bullying without defining it, can lead to different results when the same population is surveyed using an instrument such as the NAQ-R that specifically defines the term. This demonstrates the importance of using a specific definition, ideally a standard definition, for studies of this phenomenon.

Some results of studies of bullying have varied from country to country and merit further investigation. For example, our study finds that those nurses who had worked longer in the ED reported experiencing less bullying. This result is consistent with those of ALBashtawy, et al., who found, also in Jordan, that workers in the ED who are over 30 years old are less likely to experience violent incidents [23]. However, the opposite result was observed by Johnson and Rea in the U.S. [40]. As another example, varying results have been found in studies that have examined bullying in relation to shifts worked by nurses. In our study, nurses who worked in rotating shifts reported a slightly higher prevalence of bullying acts than nurses on the day shift. This same association was found in two studies from Asia [29, 38], and one study from New York [46], but not in a third, from Europe [47], which reported that nurses working on the A (day) shift are prone to more aggressive behaviors and bullying. In our study, most WPB incidences occurred at the B shift (from 3 pm to 11 pm) and this seems to be related to the following factors: the absence of administrative personnel, work pressure, inadequate staffing, and the increased access of public during this time after the outpatient clinics close their doors and leave patients with no choice other than the EDs.

The following is an example where the research across countries, to date, has been consistent: We found that the highest percentage of the bulliers were nurses working in the same unit (30.0%). This is similar to results reported from the southern United States and Turkey [27, 37]. It is possibly related to the number and type of nurses in a unit. The number of nurse co-workers will be more than the number of physicians, and the number of physicians will be more than the number of charge nurses.

### Limitations

Our study uses self-reported data collection instruments. Thus, we can evaluate nurses’ reports and perceptions; but we do not have data from direct observations of the nurses while they are at work. Additionally, we used only bivariate data analysis. Our data applied to the five studied hospitals in Amman; and, we believe they are likely to be generalizable to all hospitals in Jordan. This study did not specify a theoretical framework or a conceptual model. Rather, this was an exploratory analysis of the occurrence of WPB in Jordanian EDs and of factors that might lead to prevention. Possibilities for future studies include a larger sample of nurses to identify the most important predictors of bullying. Future studies in Jordan should also include measures of bullying predictors that we did not include in the present study. Moreover, future studies might include a larger sample of nurses and include multivariate analyses to identify the most important predictors of bullying.

### Implications for nursing practice and policy

We believe that to decrease the occurrence of WPB in hospitals, the organization must develop training programs for nurses and their leaders that include anger management, conflict management, and improvement of communication skills. There should be explicit institutional policies that cover workplace bullying and violence; and reporting of all incidents should be encouraged. On a national basis, creating specific laws on safety of nurses should be considered. The combination of legislation, institutional policy, education, and practical support can help enable nurses to provide care in a bullying-free environment. We firmly believe that this is important for promoting better quality of care.

## Conclusion

Bullying behavior in the workplace is harmful. It affects employees, the organizations they work in, and the clients or patients they serve. This study interestingly documents a high incidence of WBP and that the main perpetrators and victims of bullying were male nurses, which is not the population found in western countries. Based on this result we can conclude that “bullies” are not simply one gender towards the opposite. Our study supports the concept that WPB does affect an employee’s productivity, and this ultimately affects an organization’s productivity. Most importantly, in healthcare settings, WBP ultimately affects quality of care. This makes a compelling argument for the need to focus on its prevention. We strongly recommend that every healthcare institution develop and implement policies and practices that will minimize workplace bullying and violence.
